# Enrichment of Large-Diameter Single-Walled Carbon Nanotubes (SWNTs) with Metallo-Octaethylporphyrins

**DOI:** 10.3390/ma6083064

**Published:** 2013-07-24

**Authors:** Yuda Li, A. F. M. Mustafizur Rahman, Gang Liu, Zichao Xiong, Kenji Koezuka, Zhigao Xu, Naoki Komatsu, Feng Wang

**Affiliations:** 1 Key Laboratory for Green Chemical Process of Ministry of Education, Wuhan Institute of Technology, Wuhan 430073, China; E-Mails: psydlee@163.com (Y.L.); pszcxiong@163.com (Z.X.); xuzhigaowit@126.com (Z.X.); 2 Department of Applied Chemistry and Chemical Engineering, University of Dhaka, Dhaka 1000, Bangladesh; E-Mail: banglatapu@yahoo.com; 3 Department of Chemistry, Shiga University of Medical Science, Seta, Otsu 520-2192, Japan; E-Mails: gliu@belle.shiga-med.ac.jp (G.L.); kokonattu101304@yahoo.co.jp (K.K.); nkomatsu@belle.shiga-med.ac.jp (N.K.)

**Keywords:** single-walled carbon nanotubes, metallo-octaethylporphyrins, complexation, enrichment

## Abstract

We report here a detailed experimental investigation on noncovalent functionalization of single-walled carbon nanotubes (SWNTs) with four different metallo-octaethylporphyrins (MOEPs). It has been found that the identity of the center metal of MOEP strongly influences the solubilization of SWNTs. MnOEPs and ZnOEPs successfully extracted SWNTs in methanol, as confirmed by absorption spectroscopy, while CoOEPs and CuOEPs were not able to extract SWNTs at all. Atomic force microscopy (AFM) studies revealed that large SWNTs bundles could be exfoliated into either individual SWNTs or very small bundles by complexation with ZnOEP molecules. As for enrichment of SWNTs, ZnOEPs and MnOEPs show similar diameter discrimination ability toward 76-CoMoCAT, providing the extracted SWNTs with relatively large diameters.

## 1. Introduction

The unique physical and chemical properties of single-walled carbon nanotubes (SWNTs) have promised much potential for a vast range of device applications, such as field-effect transistors [1], photovoltaic [2,3], field-emission display [4] and chemical sensors [5,6]. The present state of the technology for SWNTs synthesis always produced samples with various kinds of structures. As the optical and electronic properties of SWNTs are determined by their diameters and roll-up indices (*n*,*m*), structural control of SWNTs is important for their applications [7]. Therefore, various methods of selective separation and purification of SWNTs with specific structural properties have been extensively investigated over the last decade [8,9,10,11,12,13,14,15,16,17,18,19,20,21,22,23,24,25,26].

In the past few years, the functionalization of SWNTs with aromatic molecules, such as pyrene, perylene, porphyrin and their derivatives, had drawn broad attention, because of their strong affinity toward the aromatic sidewalls of SWNTs through π–π interaction [27]. Among the aromatic molecules, porphyrins are the most extensively studied, and the interest has been further expanded to porphyrin/SWNTs nanocomposites, due to their excellent photophysical [28,29], electronic [30,31] and optical properties [32]. Nakashima *et al.* first reported that Zn-protoporphyrins (ZnPPs) can form stable complexes with SWNTs in *N*,*N*-dimethylformamide (DMF) [33]. Although the metal-free 2,3,7,8,12,13,17,18-octaethyl-21H,23H-porphyrin (OEP) and ZnOEP were found to be good SWNT solubilizers, no obvious discrimination of diameter of SWNTs was observed [34]. Previous studies have concluded that free base tetraphenylporphyrin (TPP) derivatives [35] and metallo-tetraphenyl porphyrins [36] containing Zn(II), Mn(III), Co(II) and Cu(II) have been shown to selectively interact with semiconducting SWNTs. A theoretical approach to comprehensively understand the π–π interaction between porphyrins and SWNTs has also been reported [37]. In our previous work, we have reported that chiral Zn–porphyrins can serve as significant host molecules for separating SWNTs, providing optically active ones with limited (*n*,*m*) structures [38,39,40,41,42].

**Figure 1:**
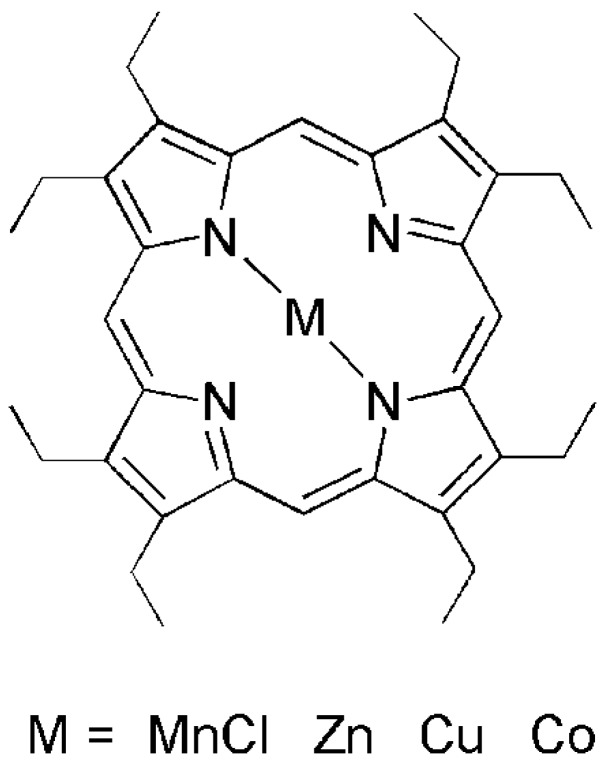
Chemical structure of metallo-octaethylporphyrin (MOEP).

The supramolecular metalloporphyrin-SWNT system has quite interesting magnetic, electronic and optical properties with broad applications, such as biosensors [43], solar energy conversion [44] and field-effect transistors [36]. Obviously, it is important to understand the π–π interaction between metalloporphyrin molecules and SWNTs in detail to make further progress in the application of such complexes. In this study, we report the complexation of SWNTs with metallo-octaethylporphyrin (MOEP) molecules containing Mn(III), Co(II), Cu(II) and Zn(II) and discuss their extraction ability (Figure 1). To the best of our knowledge, the diameter discrimination of manganese porphyrin toward SWNTs has not been reported before. We believe that our present results are useful to study the π–π interaction between metalloporphyrin molecules and SWNTs from an experimental point of view.

## 2. Results and Discussion

### 2.1. Supramolecular Interactions of MOEP with 76-CoMoCAT

All the four metalloporphyrins displayed good solubility in organic solvents, such as chloroform, dichloromethane, toluene, tetrahydrofuran (THF) and acetone. However, no SWNTs were extracted in the presence of the metalloporphyrins, indicating no significant interaction between the porphyrins and SWNTs in the solvents mentioned above. Similar phenomena have been observed in the case of diporphyrin nanotweezers and dipyrene nanotweezers [22,38,39,40,41,42]. In our previous work, methanol was chosen as the appropriate solvent to dissolve the SWNT complex. Unfortunately, neither CuOEP nor CoOEP was able to extract 76-CoMoCAT in methanol. Instead, they were found to be extracted in the solid phase. After the porphyrins were recovered from the complexes by pyridine and THF washing, CuOEP and CoOEP species were found to be present at a significantly lower amount in the corresponding porphyrin/SWNT complexes, suggesting that due to a weaker interaction, they are not able to de-bundle and disperse the SWNTs. In other word, in the CuOEP/SWNT or CoOEP/SWNT complexes, the porphyrin content is rather lower compared to that of SWNTs (with an approximate proportion of 1:12 and 1:11, respectively), which might account for the poor extraction ability for MOEPs mentioned above in methanol. When the same experiment was conducted by use of ZnOEP, a very dark suspension was obtained with an absorbance of 1.9 at a wavelength of 851 nm in a 1 cm cell, which corresponds to SWNTs solubility reaching close to 0.1 mg/mL [45]. After centrifugation at 50,400 × *g* for 3 h, the resulting supernatant remained very stable with no sedimentation for several months. The highest ZnOEP content with a proportion of roughly 1:1 was found in the ZnOEP/SWNT complexes, indicating that ZnOEPs are capable of intercalating the bundles of SWNTs and, therefore, fully covering the surface of SWNTs, because of the strong π–π interaction between ZnOEPs and SWNTs. In addition to their π–π interaction, efficient charge transfer interaction between electron-deficient SWNTs with n-donor ZnOEPs might increase the polarity of the complex and, therefore, contribute to the formation of very stable and highly soluble SWNT complexes in better solvent, such as methanol [22], preventing re-bundling of the SWNT complex with ZnOEP [33]. In contrast, when MnOEP was used, a small amount of SWNTs were collected from the extracts under milder conditions of centrifugation (15,000 × *g* for 30 min). After concentration of the supernatant, the residue was washed with pyridine and THF to thoroughly remove the porphyrins. Zero-point-three milligrams and 0.05 mg of SWNTs were recovered from the complexes with ZnOEP and MnOEP, respectively. Although we cannot provide more detailed evidences for such a significant difference in their extraction ability, so far, we think that the difference may be due to the different interaction between different metalloporphyrin molecules and SWNTs. As shown in Figure 1, these four metalloporphyrin samples exhibit quite a similar molecular structure. The strongest interaction with SWNTs occurs in the case of ZnOEP, which is able to fully exfoliate and considerably stabilize SWNT bundles and, therefore, results in the dispersion with the highest SWNT concentration. In contrast, CuOEP and CoOEP are unable to do so, because of a weaker interaction and, therefore, are only capable of attaching to the sidewalls of SWNT bundles. As a result, we might conclude that an appropriate central metal atom, such as zinc and manganese, helps increase the interaction between porphyrins and SWNTs and, therefore, solubilizes the complexes of ZnOEP and MnOEP in methanol, while metal-ion, such as Cu(II) and Co(II), incorporated into porphyrin may decrease the solubility of the SWNT complexes of CoOEP and CuOEP in methanol.

**Figure 2:**
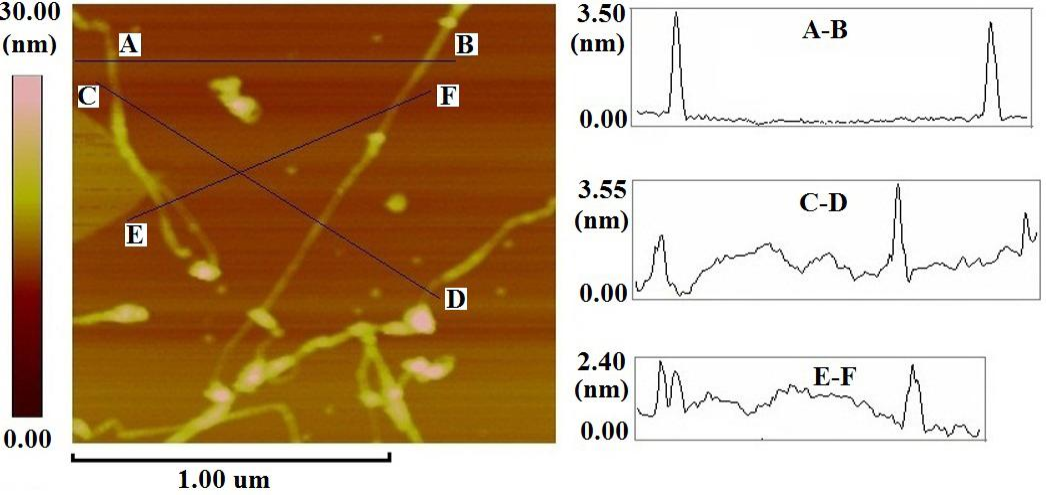
Atomic force microscopy (AFM) image of single-walled carbon nanotubes (SWNTs) complexes of ZnOEP with 76-CoMoCAT on freshly cleaved mica. z-Profiles along A–B, C–D and E–F in the AFM image.

Additional characterization of the complexes of ZnOEP with 76-CoMoCAT was performed using atomic force microscopy (AFM). The AFM image of the ZnOEP-SWNT sample (Figure 2), prepared by casting one drop of the extract onto a freshly cleaved mica substrate, clearly shows many SWNT strings. Molecular aggregates of excess ZnOEPs are also seen to be spread across the substrate. Height analysis of these features suggests that most of the SWNT strings ranged in height from *ca*. 2.0 to *ca.* 5.0 nm. Since the diameter of 76-CoMoCAT SWNTs is approximately 0.9 nm, the heights of 2.0 and 5.0 nm should correspond to the individual SWNTs covered with ZnOEP and small bundles of porphyrin-coated SWNTs, respectively. This image, again, demonstrates that ZnOEPs strongly interact with SWNTs and are able to exfoliate them into individual SWNTs or small bundles of SWNTs in solution.

### 2.2. Absorption Spectra of the Extracts

**Figure 3:**
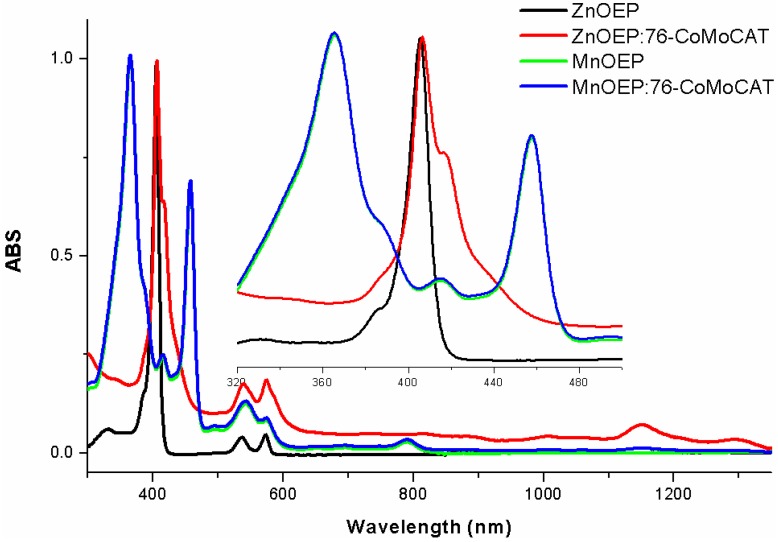
UV-Vis–NIR spectra of ZnOEP and MnOEP in methanol and the supernatant after extraction of 76-CoMoCAT with ZnOEP and MnOEP in methanol. The spectra were normalized at 410 nm. Inset: expansion of the spectra in the regions of 320–500 nm.

Figure 3 depicts ultra-violet visible near infrared (UV-Vis–NIR) spectra of a homogenous solution of ZnOEP and its SWNT complex in methanol. Free ZnOEP exhibited the Soret band around 410 nm and the Q band around 550 nm. After the extraction with 76-CoMoCAT, new absorption bands appeared as broad peaks at 416, 1145 and 1280 nm. The new peak at 416 nm is an indication of π–π interaction between the porphyrins and the nanotubes. The upward shift of the base line after extraction, as shown in Figure 3, together with the black color of the supernatant suggests the existence of SWNTs in the supernatant. In the extraction with MnOEP, on the other hand, a very slight upward shift was observed in the absorption of the MnOEP-SWNT complex as compared with the free MnOEP, probably due to the very low concentration of the extracted SWNTs. The peak intensity at 1145 and 1280 nm in ZnOEP-SWNT is much higher than that in MnOEP-SWNT, which can be ascribed to the improved extraction ability of ZnOEP. These results support the above conclusion of the better SWNT extraction ability of ZnOEP compared to MnOEP.

### 2.3. Large-Diameter Enrichment of 76-CoMoCAT through Extraction

**Figure 4:**
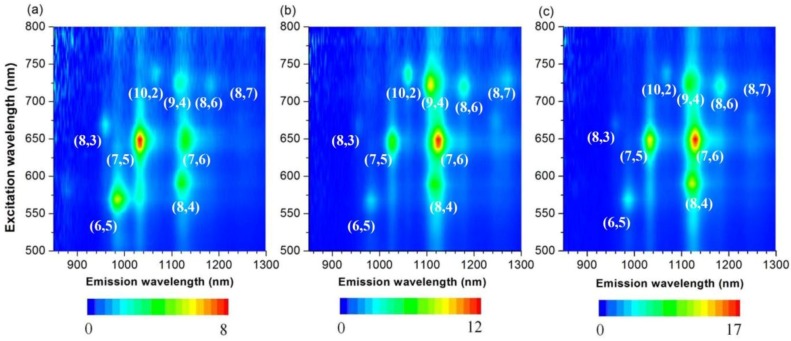
Photoluminescence spectra of D_2_O/SDBS (sodium dodecyl benzene sulfonate) solutions of 76-CoMoCAT before (**a**); and after extraction with ZnOEP (**b**); and MnOEP (**c**).

As a complementary measurement, 3D photoluminescence (PL) spectra provide comprehensive information for semiconducting SWNTs [46]. The metallic SWNTs were non-emissive and, thus, were not detected by fluorescence. The PL experimental conditions used herein cover the whole (*n*,*m*) semiconducting SWNTs with broad diameter distribution spanning from 0.76 nm ((6,5)-SWNTs) to 1.03 nm ((8,7)-SWNTs), shown in Figure 4a. In order to compare the change of the (*n*,*m*) abundance of SWNTs quantitatively, we calculated the intensity of each peak from the PL spectra, and the results are summarized in Table 1. Since different (*n*,*m*)-SWNTs have a different coefficient in absorption and PL spectroscopies, the abundance in Table 1 is not actual one, but estimated to be one. After the extraction of 76-CoMoCAT with ZnOEP, (10,2), (7,6), (9,4), (8,6) and (8,7)-SWNTs (0.88–1.03 nm in diameter) having relatively larger diameters were enriched, while the abundance of SWNTs with smaller diameters, such as (6,5), (8,3) and (7,5)-SWNTs, were decreased, as shown in the PL spectra. In particular, the peak corresponding to (8,3)-SWNTs almost disappeared. These results clearly indicate that ZnOEPs preferentially extract 76-CoMoCAT SWNTs with large diameters ranging from 0.88 to 1.03 nm. For the extraction of 76-CoMoCAT with MnOEP, the abundance of (8,4), (7,6), (9,4), (8,6) and (8,7)-SWNTs was enhanced, as shown in Figure 4a,c and Table 1. The enhanced SWNTs have diameters from 0.84 to 1.03 nm. Although the diameter discrimination ability of both the metalloporphyrin molecules are similar, the content of (8,3) and (7,5)-SWNTs is decreased more significantly in the case of ZnOEP.

**Table 1:** (*n*,*m*) Abundance estimated from photoluminescence (PL) spectra of semiconducting SWNTs before and after extraction of 76-CoMoCAT with ZnOEP and MnOEP.

Major (*n*,*m*) components in 76-CoMoCAT	Diameter (nm)	Roll-up angle (degree/°)	Abundance (%) estimated from PL spectra		
			76-CoMoCAT	Extraction with ZnOEP	Extraction with MnOEP
(6,5)	0.76	27.0	18	5	7
(8,3)	0.78	15.3	6	3	3
(7,5)	0.83	24.5	25	14	18
(8,4)	0.84	19.1	13	13	18
(10,2)	0.88	9.0	5	7	5
(7,6)	0.90	27.5	13	26	25
(9,4)	0.92	17.5	11	19	12
(8,6)	0.97	25.3	5	8	8
(8,7)	1.03	27.8	4	5	4

**Figure 5:**
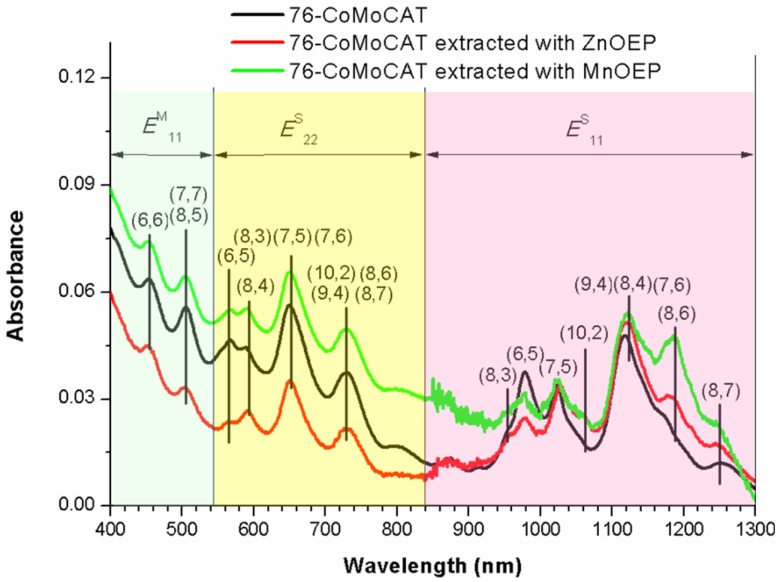
UV-Vis–NIR spectra of D_2_O/SDBS solutions of 76-CoMoCAT and the ones extracted with ZnOEP and MnOEP.

The absorption spectra shown in Figure 5 are consistent with the above conclusion of the large-diameter selectivity of ZnOEP and MnOEP. UV-Vis–NIR spectra in this study are dominated by the absorption of the *E*^S^_11_ and *E*^S^_22_ bands of the semiconducting SWNTs in the wavelength region from 542 nm to 1250 nm. After the extraction of 76-CoMoCAT with ZnOEP, a decrease in the abundance is observed for (8,3), (6,5) and (7,5)-SWNTs in the *E*^S^_11_ region of the absorption spectra (red trace in Figure 5). In contrast, the absorption peaks of (10,2), (8,4), (7,6), (9,4) and (8,6)-SWNTs increase relatively. Since the absorption peaks of (8,4), (7,6) and (9,4) in the *E*^S^_11_ region are close to each other, it is very difficult to precisely distinguish their *E*^S^_11_ absorption peaks [41]. However, the peak at 590 nm corresponding to *E*^S^_22_ of (8,4)-SWNTs was slightly decreased after the extraction. These results clearly show that the content of (10,2), (7,6), (9,4) and (8,6)-SWNTs were enriched after the extraction with ZnOEP. In the case of MnOEP extraction, we observe that larger diameter SWNTs, such as (10,2), (8,4), (7,6), (9,4) and (8,6)-SWNTs, were extracted preferentially (green trace in Figure 5), and the selectivity toward diameter of SWNTs is quite similar to that of ZnOEP.

**Figure 6:**
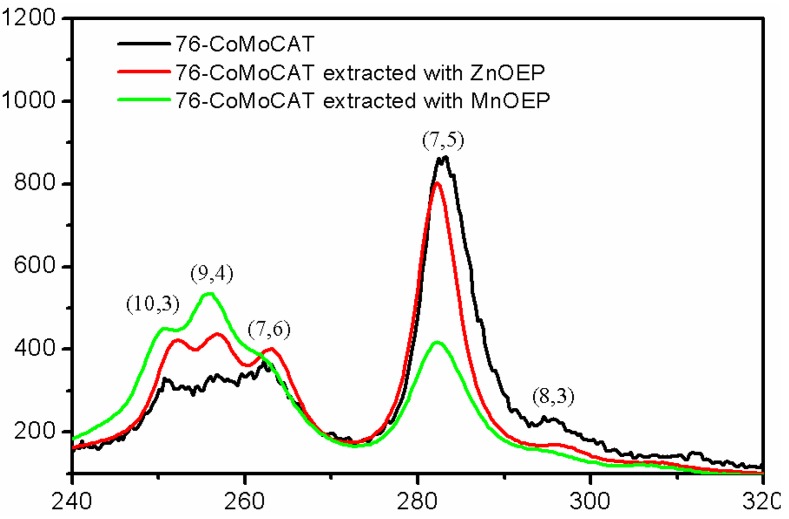
Raman spectra of 76-CoMoCAT before and after extraction with ZnOEP and MnOEP at an excitation wavelength of 633 nm. The Raman spectra were normalized to the G-band intensity.

In addition to the PL and absorption spectra, we also measured SWNTs with Raman spectroscopy, which is a powerful tool for the characterization of SWNTs, namely, and their diameters. Raman spectra of solid samples of 76-CoMoCAT SWNTs and the extracted SWNTs after removal of MOEPs were measured at the excitation wavelength of 633 nm, as shown in Figure 6. The relative abundance of SWNTs in Table 2 was determined by deconvolution of the Raman spectra (Figure 6), shown in Figure S1 in the Supporting Information. Since Raman measurement exhibits some limitations for a reliable quantitative assessment of SWNTs purity, due to its sensitivity to excitation wavelength, temperature and degree of bundling, we calculated the relative content of the identified semiconducting SWNTs based on Raman intensity before and after the extraction. In the radial breathing mode (RBM), (7,6), (9,4) and (10,3)-SWNTs with relatively larger diameters (0.88, 0.92 and 0.94 nm, respectively) increased, and accordingly, the content of (7,5) and (8,3)-SWNTs with the smaller diameter decreased on the basis of the intensity of the G-band, supporting the diameter selectivity toward 76-CoMoCAT SWNTs mentioned above.

**Table 2:** The relative content of semiconducting SWNTs based on Raman intensity before and after the extraction of 76-CoMoCAT with ZnOEP and MnOEP.

Major ( *n*,*m*) components in 76-CoMoCAT	Diameter (nm)	Roll-up angle (degree/°)	Abundance (%) estimated from Raman spectra		
			76-CoMoCAT	Extraction with ZnOEP	Extraction with MnOEP
(8,3)	0.78	15.3	5.8	1.6	2
(7,5)	0.83	24.5	57.9	48.6	27.3
(7,6)	0.90	27.5	15.7	16.2	19.4
(9,4)	0.92	17.5	8.4	17.3	27.2
(10,3)	0.94	12.7	12.1	16.3	24.1

**Figure 7:**
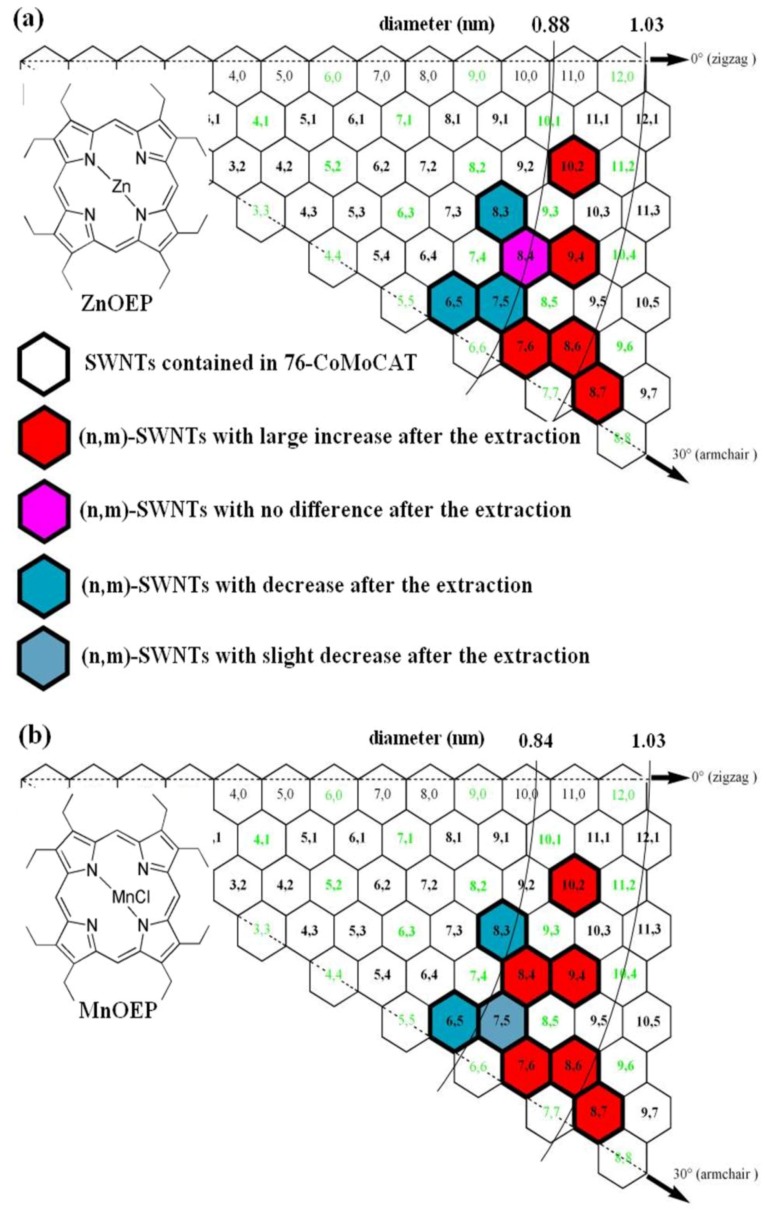
Summary of enrichment in (*n*,*m*) abundance in the extraction of 76-CoMoCAT with ZnOEP (**a**); and MnOEP (**b**).

The results of the (*n*,*m*) enhancements in semiconducting SWNTs through the extraction with ZnOEP and MnOEP are summarized in the graphene map shown in Figure 7a,b, respectively. The map reveals that (10,2), (7,6), (9,4), (8,6) and (8,7)-SWNTs having similar diameters in the range of 0.88–1.03 nm are enhanced after the extraction with ZnOEP (Figure 7a). As compared with ZnOEP, MnOEP exhibits similar discrimination ability towards the diameters of SWNTs, as shown in Figure 7b.

**Figure 8:**
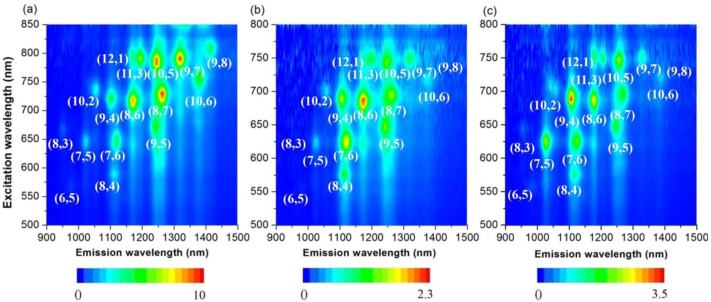
Photoluminescence spectra of D_2_O/SDBS solutions of HiPCO before (**a**); and after extraction with ZnOEP (**b**) and MnOEP (**c**).

For comparison, we have also studied the extraction process by using HiPCO (high pressure carbon monoxide decomposition process) SWNTs (with a wider diameter distributions compared with 76-CoMoCAT SWNTs) and the results support the above conclusion of the diameter selectivity of MOEPs. Figure 8 shows the PL spectra for HiPCO SWNTs dispersed in D_2_O with the aid of SDBS before and after extraction with porphyrins. Similar analysis for the abundance of each species is listed in Table S1. In the PL spectra of HiPCO extracted with ZnOEP (Figure 8b), the content of SWNTs with a diameter larger than 1.03 nm, for example (10,5), (9,7), (10,6) and (9,8)-SWNTs, decreased relatively. The content of (7,5)-SWNTs with 0.83 nm in diameter is almost the same before and after the extraction. In contrast to the extraction with ZnOEP, (7,5)-SWNTs were largely enhanced after the solubilization by MnOEP, although the content of the SWNTs with a diameter larger than 1.03 nm decreased similarly, as shown in Figure 8c.

**Figure 9:**
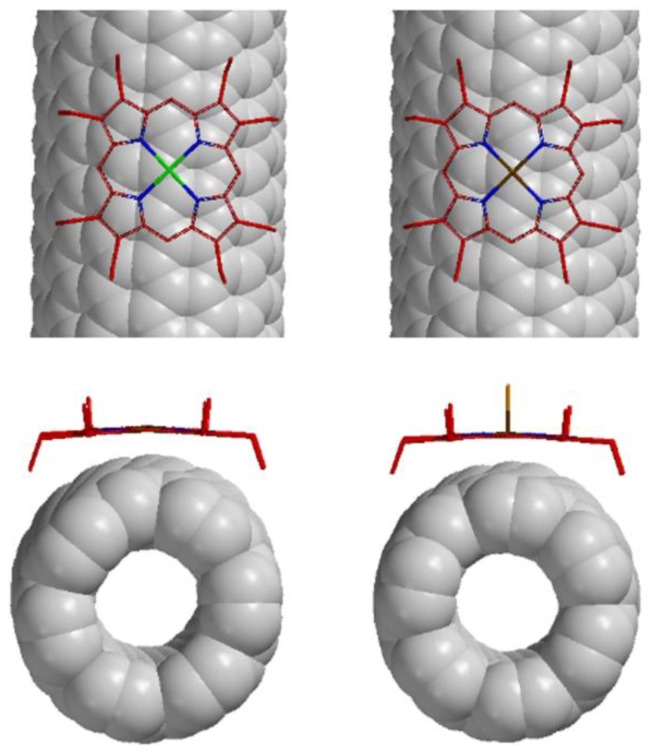
Computer-generated molecular modeling of the complex structures: side views (top) and views along the SWNT axis (bottom) of 1:1 complexes of ZnOEP and (7,6)-SWNT (left) and MnOEP and (7,6)-SWNT (right).

The computer-generated molecular models of the complexes between (7,6)-SWNT and MOEP with the use of the Spartan program (Wavefunction Inc., Irvine, CA, USA) show that the porphyrin adsorbs strongly on the surface of SWNTs, due to the π–π interaction between them (Figure 9), and the strength of the stacking interaction probably depends on the area of the overlap in these two π systems [40] and the closet distance between the center metal and the surface of SWNTs [37]. Since the two complexes—ZnOEP: (7,6)-SWNT and MnOEP: (7,6)-SWNT—exhibited the quite similar overlapped area and the closet M–tube distance, respectively, we think that the two π–π stacking interactions existing in the complexes of (7,6)-SWNT and ZnOEP and (7,6)-SWNT and MnOEP are almost the same, which suggest that ZnOEP and MnOEP show a similar preference in SWNT diameter.

## 3. Experimental Section

### 3.1. Materials

Samples of SWNTs grown by both the “CoMoCAT” (designated as 76-CoMoCAT in this paper) [47] and “HiPCO” processes were purchased from Sigma-Aldrich Co. and Carbon Nanotech., respectively. 2,3,7,8,12,13,17,18-octaethyl-21H,23H-porphyrin manganese(III) chloride (MnOEP), and the zinc(II), copper(II) and cobalt(II) analogues (ZnOEP, CuOEP and CoOEP, respectively) were purchased from Sigma-Aldrich Co. Chemical structure of the metalloporphyrin is shown in Figure 1. All the reagents, unless otherwise specified, were obtained from Sigma-Aldrich Co., Tokyo, Japan, Wako Pure Chemical Industries and Tokyo Chemical Industry Co., Ltd., Tokyo, Japan, and were used as received.

### 3.2. Equipment

UV-Vis–NIR absorption spectra were recorded on a UV-3100PC scanning spectrophotometer (Shimadzu Co., Kyoto, Japan). Photoluminescence (PL) spectra were measured on a NIR-PL system (Shimadzu Co., Tokyo, Japan). Raman spectral measurements were conducted using LabRam HR800 (Horiba Ltd., Kyoto, Japan). Atomic force microscopy (AFM) imagery was recorded in tapping mode with a Multimode Nanoscope IIIa microscope (Veeco Inc., Santa Barbara, CA, USA) using a Si cantilever of resonant frequency 260 kHz and a force constant of 60 N m^−1^. Centrifugation was carried out with a Beckman Avanti J-E, Optima-TL and L-70. Tip-sonication was performed with MISONIX (80 W, 20 kHz).

### 3.3. Extraction of 76-CoMoCAT with ZnOEP and MnOEP

76-CoMoCAT (10 mg) and ZnOEP or MnOEP (10 mg) in methanol (40 mL) were bath-sonicated at 20 °C for 24 h. The obtained suspension was subjected to centrifugation at 50,400 × *g* for 3 h for the extraction with ZnOEP and centrifugation at 15,000 × *g* for 30 min for the extraction with MnOEP, respectively. The homogeneous supernatant was subjected to UV-Vis–NIR measurements (Figure 3). After concentration of the supernatant, the residue was washed with pyridine and THF several times, until the porphyrin Soret band disappeared in the UV-Vis spectra of the washings. The extracted SWNTs free from metalloporphyrins and 76-CoMoCAT SWNTs were analyzed by Raman spectroscopy (Figure 6) and were sonicated in D_2_O (18.5 mL) with a tip-type apparatus in the presence of SDBS (10 mg/mL) for 40 min. The resulting solution was centrifuged at 136,000 × *g* for 30 min, and the supernatant (upper 80%) was subjected to PL (Figure 4) and UV-Vis–NIR (Figure 5) measurements.

## 4. Conclusions

In this work, we investigate the functionalization of 76-CoMoCAT SWNTs with commercial metalloporphyrin. The extraction ability of SWNTs has been found to be largely dependent on the identity of the center metal of metalloporphyrin. ZnOEPs were able to extract large amounts of SWNTs, and MnOEPs were found to have less extraction ability of SWNTs, whereas CoOEPs and CuOEPs did not show any solubility to 76-CoMoCAT. As for enrichment of semiconducting SWNTs, ZnOEP successfully extracted large-diameter 76-CoMoCAT SWNTs with a diameter range of 0.88–1.03 nm, and the (*n*,*m*) enhanced by MnOEP possesses a similar diameter range (0.84–1.03 nm). So far, we cannot provide a detailed explanation for such a similar behavior of ZnOEP and MnOEP; it has probably to do with the strength of the stacking interaction between SWNT and metalloporphyrin. Studies toward an understanding of the mechanisms of separation of SWNTs upon complexation with metalloporphyrins are ongoing in our group.

## Supplementary Materials

Supplementary materials can be accessed at: http://www.mdpi.com/1996-1944/6/8/3064/s1.

## References

[1] Artukovic E., Kaempgen M., Hecht D.S., Roth S., Grüner G. (2005). Transparent and flexible carbon nanotube transistors. Nano Lett..

[2] Rowell M.W., Topinka M.A., McGehee M.D., Prall H.-J., Dennler G., Sariciftci N.S., Hu L., Gruner G. (2006). Organic solar cells with carbon nanotube network electrodes. Appl. Phys. Lett..

[3] Schuettfort T., Nish A., Nicholas R.J. (2009). Observation of a type II heterojunction in a highly ordered polymer−carbon nanotube nanohybrid structure. Nano Lett..

[4] Zhang D., Ryu K., Liu X., Polikarpov E., Ly J., Tompson M.E., Zhou C. (2006). Transparent, conductive, and flexible carbon nanotube films and their application in organic light-emitting diodes. Nano Lett..

[5] Pang X., Imin P., Zhitomirsky I., Adronov A. (2010). Amperometric detection of glucose using a conjugated polyelectrolyte complex with single-walled carbon nanotubes. Macromolecules.

[6] Wang F., Gu H., Swager T.M. (2008). Carbon nanotube/polythiophene chemiresistive sensors for chemical warfare agents. J. Am. Chem. Soc..

[7] Rao C.N.R., Voggu R., Govindaraj A. (2009). Selective generation of single-walled carbon nanotubes with metallic, semiconducting and other unique electronic properties. Nanoscale.

[8] Backes C., Mundloch U., Ebel A., Hauke F., Hirsch A. (2010). Dispersion of hipco^®^ and comocat^®^ single-walled nanotubes (swnts) by water soluble pyrene derivatives—depletion of small diameter swnts. Chem. A Eur. J..

[9] Backes C., Schmidt C.D., Hauke F., Hirsch A. (2011). Perylene-based nanotweezers: Enrichment of larger-diameter single-walled carbon nanotubes. Chem. A Eur. J..

[10] Chen F., Wang B., Chen Y., Li L.-J. (2007). Toward the extraction of single species of single-walled carbon nanotubes using fluorene-based polymers. Nano Lett..

[11] Ghosh S., Bachilo S.M., Weisman R.B. (2010). Advanced sorting of single-walled carbon nanotubes by nonlinear density-gradient ultracentrifugation. Nat. Nano.

[12] Hersam M.C. (2008). Progress towards monodisperse single-walled carbon nanotubes. Nat. Nano.

[13] Hwang J.-Y., Nish A., Doig J., Douven S., Chen C.-W., Chen L.-C., Nicholas R.J. (2008). Polymer structure and solvent effects on the selective dispersion of single-walled carbon nanotubes. J. Am. Chem. Soc..

[14] Kalbác M., Kavan L., Dunsch L. (2009). Selective etching of thin single-walled carbon nanotubes. J. Am. Chem. Soc..

[15] Kato Y., Niidome Y., Nakashima N. (2009). Efficient separation of (6,5) single-walled carbon nanotubes using a “nanometal sinker”. Angew. Chem. Int. Ed..

[16] Komatsu N., Wang F. (2010). A comprehensive review on separation methods and techniques for single-walled carbon nanotubes. Materials.

[17] Krupke R., Hennrich F., Löhneysen H.V., Kappes M.M. (2003). Separation of metallic from semiconducting single-walled carbon nanotubes. Science.

[18] Liu H., Nishide D., Tanaka T., Kataura H. (2011). Large-scale single-chirality separation of single-wall carbon nanotubes by simple gel chromatography. Nat. Commun..

[19] Marquis R., Greco C., Sadokierska I., Lebedkin S., Kappes M.M., Michel T., Alvarez L., Sauvajol J.-L., Meunier S.P., Mioskowski C. (2008). Supramolecular discrimination of carbon nanotubes according to their helicity. Nano Lett..

[20] Marquis R., Kulikiewicz K., Lebedkin S., Kappes M.M., Mioskowski C., Meunier S., Wagner A. (2009). Axially chiral facial amphiphiles with a dihydronaphthopentaphene structure as molecular tweezers for swnts. Chem. A Eur. J..

[21] Nish A., Hwang J.-Y., Doig J., Nicholas R.J. (2007). Highly selective dispersion of single-walled carbon nanotubes using aromatic polymers. Nat. Nano.

[22] Rahman A.F.M.M., Wang F., Matsuda K., Kimura T., Komatsu N. (2011). Diameter-based separation of single-walled carbon nanotubes through selective extraction with dipyrene nanotweezers. Chem. Sci..

[23] Stürzl N., Hennrich F., Lebedkin S., Kappes M.M. (2009). Near monochiral single-walled carbon nanotube dispersions in organic solvents. J. Phys. Chem. C.

[24] Tu X., Manohar S., Jagota A., Zheng M. (2009). DNA sequence motifs for structure-specific recognition and separation of carbon nanotubes. Nature.

[25] Zhang L., Tu X., Welsher K., Wang X., Zheng M., Dai H. (2009). Optical characterizations and electronic devices of nearly pure (10,5) single-walled carbon nanotubes. J. Am. Chem. Soc..

[26] Zheng M., Semke E.D. (2007). Enrichment of single chirality carbon nanotubes. J. Am. Chem. Soc..

[27] Zhao Y.-L., Stoddart J.F. (2009). Noncovalent functionalization of single-walled carbon nanotubes. Acc. Chem. Res..

[28] Cheng F., Zhu J., Adronov A. (2011). Supramolecular functionalization of single-walled carbon nanotubes with triply fused porphyrin dimers: A study of structure–property relationships. Chem. Mater..

[29] Guldi D.M., Rahman G.M.A., Jux N., Tagmatarchis N., Prato M. (2004). Integrating single-wall carbon nanotubes into donor–acceptor nanohybrids. Angew. Chem..

[30] Rahman G.M.A., Guldi D.M., Campidelli S., Prato M. (2006). Electronically interacting single wall carbon nanotube-porphyrin nanohybrids. J. Mater. Chem..

[31] Tanaka H., Yajima T., Matsumoto T., Otsuka Y., Ogawa T. (2006). Porphyrin molecular nanodevices wired using single-walled carbon nanotubes. Adv. Mater..

[32] Cheng F., Adronov A. (2006). Noncovalent functionalization and solubilization of carbon nanotubes by using a conjugated Zn–porphyrin polymer. Chem. A Eur. J..

[33] Murakami H., Nomura T., Nakashima N. (2003). Noncovalent porphyrin-functionalized single-walled carbon nanotubes in solution and the formation of porphyrin–nanotube nanocomposites. Chem. Phys. Lett..

[34] Murakami H., Nakamura G., Nomura T., Miyamoto T., Nakashima N. (2007). Noncovalent porphyrin-functionalized single-walled carbon nanotubes: Solubilization and spectral behaviors. J. Porphyr. Phthalocyanines.

[35] Li H., Zhou B., Lin Y., Gu L., Wang W., Fernando K.A.S., Kumar S., Allard L.F., Sun Y.-P. (2004). Selective interactions of porphyrins with semiconducting single-walled carbon nanotubes. J. Am. Chem. Soc..

[36] Kauffman D.R., Kuzmych O., Star A. (2007). Interactions between single-walled carbon nanotubes and tetraphenyl metalloporphyrins: Correlation between spectroscopic and fet measurements. J. Phys. Chem. C.

[37] Zhao J.-X., Ding Y.-H. (2008). Functionalization of single-walled carbon nanotubes with metalloporphyrin complexes: A theoretical study. J. Phys. Chem. C.

[38] Peng X., Komatsu N., Bhattacharya S., Shimawaki T., Aonuma S., Kimura T., Osuka A. (2007). Optically active single-walled carbon nanotubes. Nat. Nano.

[39] Peng X., Komatsu N., Kimura T., Osuka A. (2007). Improved optical enrichment of swnts through extraction with chiral nanotweezers of 2,6-pyridylene-bridged diporphyrins. J. Am. Chem. Soc..

[40] Peng X., Wang F., Kimura T., Komatsu N., Osuka A. (2009). Optical resolution and diameter-based enrichment of single-walled carbon nanotubes through simultaneous recognition of their helicity and diameter with chiral monoporphyrin. J. Phys. Chem. C.

[41] Wang F., Matsuda K., Rahman A.F.M.M., Kimura T., Komatsu N. (2011). Improved selectivity in discriminating handedness and diameter of single-walled carbon nanotubes with n-substituted 3,6-carbazolylene-bridged chiral diporphyrin nanotweezers. Nanoscale.

[42] Wang F., Matsuda K., Rahman A.F.M.M., Peng X., Kimura T., Komatsu N. (2010). Simultaneous discrimination of handedness and diameter of single-walled carbon nanotubes (swnts) with chiral diporphyrin nanotweezers leading to enrichment of a single enantiomer of (6,5)-swnts. J. Am. Chem. Soc..

[43] Tu W., Lei J., Ju H. (2009). Functionalization of carbon nanotubes with water-insoluble porphyrin in ionic liquid: Direct electrochemistry and highly sensitive amperometric biosensing for trichloroacetic acid. Chem. A Eur. J..

[44] Guldi D.M., Rahman G.M.A., Prato M., Jux N., Qin S., Ford W. (2005). Single-wall carbon nanotubes as integrative building blocks for solar-energy conversion. Angew. Chem..

[45] Wei L., Wang B., Wang Q., Li L.-J., Yang Y., Chen Y. (2008). Effect of centrifugation on the purity of single-walled carbon nanotubes from MCM-41 containing cobalt. J. Phys. Chem. C.

[46] Luo Z., Pfefferle L.D., Haller G.L., Papadimitrakopoulos F. (2006). (*n*,*m*) abundance evaluation of single-walled carbon nanotubes by fluorescence and absorption spectroscopy. J. Am. Chem. Soc..

[47] Bachilo S.M., Balzano L., Herrera J.E., Pompeo F., Resasco D.E., Weisman R.B. (2003). Narrow (*n*,*m*)-distribution of single-walled carbon nanotubes grown using a solid supported catalyst. J. Am. Chem. Soc..

