# Supplementary material for: Enrichment of Large-Diameter Single-Walled Carbon Nanotubes (SWNTs) with Metallo-Octaethylporphyrins
Source: Materials (Basel). 2013 Jul 24;6(8):3064–78. doi: 10.3390/ma6083064 (PMC5521234; doi:10.3390/ma6083064)
Supplement: Supplementary file 1 [file materials-06-03064-s001.pdf]

Materials **2013**, *6*, 3064-3078; doi:10.3390/ma6083064

OPEN ACCESS

materials

ISSN 1996-1944

www.mdpi.com/journal/materials

Article

Supplementary Information

1. Deconvolution of Raman Spectra

Figure S1. Deconvolution of Raman spectra (Figure 6) of 76-CoMoCAT (**a**) before and after extraction with ZnOEP (**b**) and MnOEP (**c**).

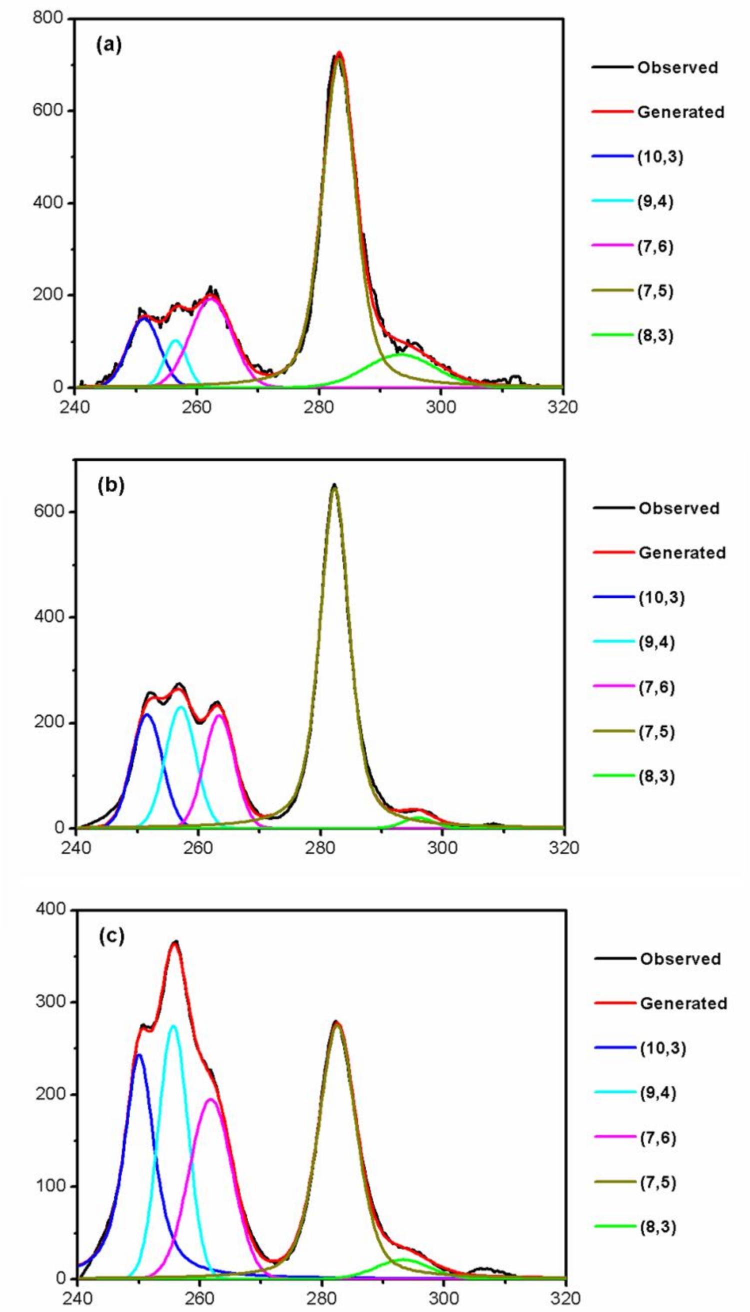

Table S1. (*n*,*m*) Abundance estimated from PL spectra of semiconducting SWNTs before and after extraction of HiPCO with ZnOEP and MnOEP.

**Major (*n*, *m*) components in HiPCO**	**Diameter (nm)**	**Roll-up angle (degree/°)**	**Abundance (%) estimated from PL spectra**		
			**HiPCO**	**Extraction with ZnOEP**	**Extraction with MnOEP**
(7,5)	0.83	24.5	2.7%	3.0%	9.5%
(10,2)	0.88	9.0	3.6%	2.9%	3.6%
(8,4)	0.84	19.1	3.5%	6.7%	5.4%
(7,6)	0.90	27.5	5.8%	11.3%	8.0%
(9,4)	0.92	17.5	6.4%	10.3%	13.8%
(8,6)	0.97	25.3	11.2%	13.8%	13.7%
(12,1)	0.99	4	5.1%	5.4%	6.3%
(11,3)	1.01	11.7	8.8%	4.8%	5.7%
(9,5)	0.98	20.6	6.9%	8.8%	6.7%
(8,7)	1.03	27.8	12.9%	9.5%	7.5%
(10,5)	1.05	19.1	11.9%	8.9%	9.8%
(9,7)	1.10	25.9	12.7%	6.7%	4.6%
(10, 6)	1.11	21.8	6.7%	4.1%	3.3%
(9,8)	1.17	28.0	5.4%	3.6%	2.2%

© 2013 by the authors; licensee MDPI, Basel, Switzerland. This article is an open access article distributed under the terms and conditions of the Creative Commons Attribution license (http://creativecommons.org/licenses/by/3.0/).
